# The effects of core stabilization exercises on the neuromuscular function of athletes with ACL reconstruction

**DOI:** 10.1038/s41598-023-29126-6

**Published:** 2023-02-07

**Authors:** Farzaneh Saki, Hossein Shafiee, Behdad Tahayori, Farzaneh Ramezani

**Affiliations:** 1 Faculty of Sports Sciences, Bu-Ali Sina University, Hamedan, Iran; 2 Department of Physical Therapy, The University of Saint Augustine for Health Sciences, Miami, FL USA

**Keywords:** Health care, Risk factors

## Abstract

Athletes who have undergone anterior cruciate ligament reconstruction (ACLR) often exhibit persistently impaired kinematics and strength. Core stability training appears to be effective for reducing high-risk landing mechanics and preventing primary anterior cruciate ligament (ACL) injuries; however, there have been few attempts to examine their effects in athletes who have undergone ACLR. This study aimed to investigate the effect of eight weeks of simple core stability training on core endurance, hip strength, and knee kinematics in ACLR athletes. Twenty-six male athletes (20–30 years old) with a history of ACL surgery with hamstring tendon autograft were randomly divided into training (n = 13) and control groups (n = 13). The training group performed core stability exercises for eight weeks before starting their team training; the control group did not receive any intervention. Both groups continued their regular team schedule. The core endurance, hip muscle strength, and knee kinematics were assessed by the McGill test, a hand-held dynamometer, and video-taping, respectively. Analysis of covariance test was used for data analysis. The training group showed a significant increase in core endurance, hip abductor and external rotator strength, knee flexion angle, and a significant decrease in the knee valgus angle during single-leg landing in post-training tests compared to their baseline tests (*P* < 0.05). Our results demonstrated that core stability exercise alters neuromuscular function to a level that is clinically acceptable and statistically significant. Because of the high incidence rate of secondary ACL injury after ACLR, it is recommended that athletes with a history of ACLR benefit from adding core stability exercises to warm-up routines or tertiary prevention programs even after completing post-operative rehabilitation. It is fast and not time-consuming to perform for athletes to reduce the risk factors of re-injury.

**Trial registration:** This study was registered in the Iranian Registry of Clinical Trials with the number IRCT20190224042827N2, registered on 19 December 2019.

## Introduction

Anterior cruciate ligament reconstruction (ACLR) is the most common form of treatment for athletes with an anterior cruciate ligament (ACL) injury and can keep an athlete out of competition for at least six months^1^. About 37% of people with ACLR do not return to their pre-injury activity level^2^ and the risk of re-injury following ACLR is more than 30%^3,4^. The incidence of secondary injury of ACLR has been estimated to be 1:4 in athletes returning to the sport, indicating a high risk of secondary injury^5^. It has been demonstrated that the neuromuscular patterns in athletes after surgery change up to two years after ACLR which may explain the high incidence rate of re-injury^6^. Therefore, to optimize the results of conventional post-operative rehabilitation, there is a need to have tertiary prevention. Having a simple tertiary prevention program during the athlete's return to sport may reduce the risk of re-injury in athlete.

Previous studies have shown that a deficit in core stability may lead to an increased risk of ACL injury^7–10^. Core stability is defined as the dynamic control of the lumbopelvic-hip complex that facilitates the transfer of torque and momentum between the lower and upper extremities during gross motor tasks of sports, exercises, and daily living^11–13^. During functional tasks, core muscles activate prior to upper and lower extremity muscles activation to produce a stable base of the extremities. In addition, the strength of the core muscles is effective in reducing the loads on the joints and in controlling the direction of the lower limbs (especially the knees)^14–16^. Lack of suitable coordination in the core muscles may lead to compensatory patterns and re-injury of the ACL^17^. A systematic review study demonstrated that poor core stability, weak hip abduction strength, increased knee valgus, and landing on heels may contribute to increased ACL injury risk in young athletes^7^. A three year prospective study demonstrated that athletes with poor core stability were less able to resist hip internal rotation moments that lead to excessive knee valgus movement during weight-bearing exercises. Therefore, these people are more likely to sustain an ACL rapture^10^. Kaji et al. (2010) examined the immediate effect of core stability exercises on postural sway. They found that short-term use of core stability exercises improves stability in the trunk, spine, and pelvis muscles and reduces postural sway^18^. A recent study conducted by Fallah Mohammadi et al. (2022) concluded that core stability exercises can improve limb symmetry in hopping task and kinetic variables during single-leg landing in patients after ACLR^19^. Attar et al. (2022) in a systematic review and meta-analysis study, investigated the effects of injury prevention programs that include core stability exercises on knee and ACL injuries. They found that exercise programs that included core stability exercises reduced the incidence of knee injuries by 46% in men and 65% in women^20^. Therefore, coordination of the core muscles is necessary for a suitable production, transmission, and control of the forces and movements that occur in the body, while weakness or decreased coordination of core muscles can lead to abnormal movement patterns and various types of sports injuries^21,22^.

Although it has been shown that core stability exercises can improve weakness and coordination of core muscles, the effects of these exercises on knee kinematics, hip strength, and trunk endurance have not yet been investigated in male athletes after ACLR. Therefore, the purpose of our study was to investigate the effects of eight weeks of simple core stability training on knee kinematics, hip strength, and trunk endurance in male athletes who had undergone ACL reconstruction and completed conventional post-operative rehabilitation. It was hypothesized that performing simple core stability training prior to team routine training could improve knee kinematics, hip strength, and trunk endurance in the training group compared with athletes who only continued their training routine.

## Methods

### Study design and participants

**Figure 1:**
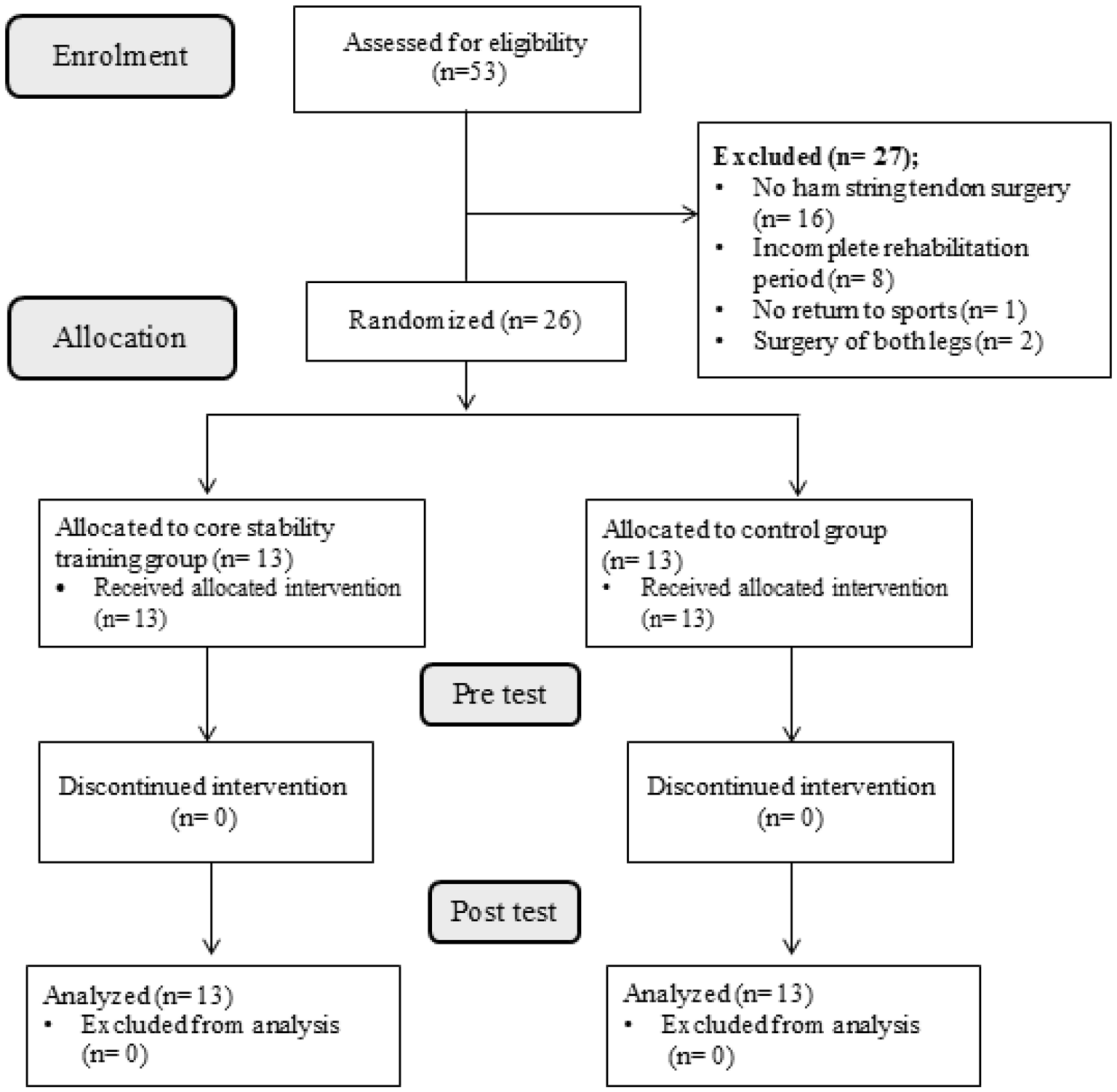
Participants Flow Diagram.

The design of this study was a randomized controlled trial with pre- and post-tests in training and control groups and was done in the sports rehabilitation laboratory of Bu-Ali Sina University. This research was single-blind in which the outcome assessor was unaware of the allocation of research groups. All participants received general information about the purpose of the study and signed the informed consent form prior to participating in the study. The study population consisted of male athletes (20–30 years) from basketball and volleyball sports disciplines who were referred to the sports medical centers in Hamedan, Iran. In the initial review of the athletes' files, 53 athletes with a history of ACLR were found. After more detailed review of the files and interviewing them, 27 people were excluded from the research, based on the inclusion and exclusion criteria (Fig. 1).

The inclusion criteria were as follows: having a history of unilateral ACL reconstruction (previous 8–12 months) using hamstring tendon autograft; completing the rehabilitation protocol with full returned to sport. The following were the exclusion criteria: having a history of ACL surgery more than once or a recurring knee problem; experiencing an injury of the trunk or lower extremity after post-operative; having a history of surgery on both legs; failing to complete the study (participation of < 80% in the sessions)^23^.

### Testing procedure

All subjects were provided and signed the consent form that was approved by the Institutional Review Board (IRB) at Bu-Ali Sina University. Basic data including age, weight, height, BMI, years of sports experience, Tegner score, and time since surgery were recorded (Table 1). Subjects were asked to do a short warm-up and then perform tests.

(i) Knee valgus and flexion angle

**Table 1:** Demographic characteristics of participants, reported as mean ± standard deviations.

	Training group (n = 13)	Control group (n = 13)	t	*P*
Age (years)	26.7 ± 2.7	25.7 ± 3.3	0.8	0.40
Height (cm)	184.0 ± 7.7	184.5 ± 6.1	-0.2	0.85
Weight (kg)	83.2 ± 9.4	81.8 ± 10.7	0.3	0.73
BMI (kg/m^2^)	24.6 ± 2.3	24.0 ± 2.5	0.6	0.54
Tegner score	6.0 ± 1.5	5.8 ± 1.5	3.9	0.69
Exercise experience (years)	13.5 ± 3.1	12.7 ± 2.3	0.8	0.44
Time since surgery (months)	8.8 ± 2.0	8.5 ± 1.7	0.5	0.61

A single-leg landing test was used to measure knee valgus (ICC = 0.94) and flexion angle (ICC = 0.98)^24^. For the single-leg landing task, athletes started from a single-legged standing position on a 30 cm high platform. Athletes stood on the healthy limb and landing on the ACLR limb. To perform this task, 6 reflective markers were placed on the ACLR limb to calculate knee valgus (Anterior Superior Iliac Spine, center of patella, and mid anterior ankle) and knee flexion angles (greater trochanter, lateral condyle of thigh, and lateral malleolus). The angle between the line of ASIS to the center of the patella and the line of center of patella to the center of the ankle was used to determine the dynamic knee valgus angle on the frontal plane, and the angle between the line of the greater trochanter to the lateral condyle of the thigh and the line of the lateral condyle to lateral malleolus was used to determine knee flexion angle in the sagittal plane^25^. Each athlete performed three trials and the mean of these three landings was used for statistical analysis^26^. No feedback was given during data collection. Knee kinematics data from the single-leg landing trials were recorded by two cameras (Fuji film hs55) in frontal view (at a distance of 366 cm) and sagittal (at a distance of 200 cm). A moderate to strong relationship has been reported between a two-dimensional and three-dimensional motion analysis for knee joint angle measurements in sagittal (0.77–0.99)^27^ and frontal (0.90–0.99)^28^ planes. Angle calculations were performed in Kinovea software.

(ii) Isometric hip muscle strength

The isometric strength of hip muscles of the male athletes with ACLR was measured using a digital hand-held dynamometer (MMT, North Coast, USA) calibrated by a one-kilogram weight before and after the measurement. The test was performed for the injured leg. Each test was performed three times for five seconds with fifteen seconds of rest between the trials. The average of the three repetitions was recorded in Kg. Finally, the value was divided by the athlete’s weight and reported as a percentage of their weight.

Hip extension tests (ICC = 0.95)^29^ were performed in prone position with knees flexed at 90°. The dynamometer was placed on the popliteal fossa as distally as possible^30^. Hip external rotators (ICC = 0.90)^29^ were tested in seated position with knees and hips flexed at 90°. The dynamometer was placed 5 cm proximal to the medial malleolus^31^. Moreover, hip abduction test (ICC = 0.85)^29^ was performed in the side-lying position with a pillow between the legs and the tested hip at approximately 0° of abduction. The dynamometer was placed 5 cm proximal to the lateral condyle^31^. Consistent verbal instructions were given to encourage the participants to make their maximal effort.

(iii) Core muscle endurance

Three isometric trunk holding tests were performed to evaluate core muscle endurance as described by McGill et al. Each test was performed once as these tests have shown to have a reliability value of > 0.97^32^. The holding time for each test until the participant’s fatigue threshold was recorded and used for the statistical analyses. Trunk flexor endurance test (ICC = 0.66)^33^ at 60 degrees was used to measure the endurance capacities of abdominal muscles. Biering-Sørensen test (ICC = 0.93)^34^ was used to measure the extensor endurance of back muscles. Lastly, side plank test (ICC = 0.95)^34^ was performed bilaterally to measure the endurance capacities of lateral core muscles^35^.

### Intervention

**Figure 2:**
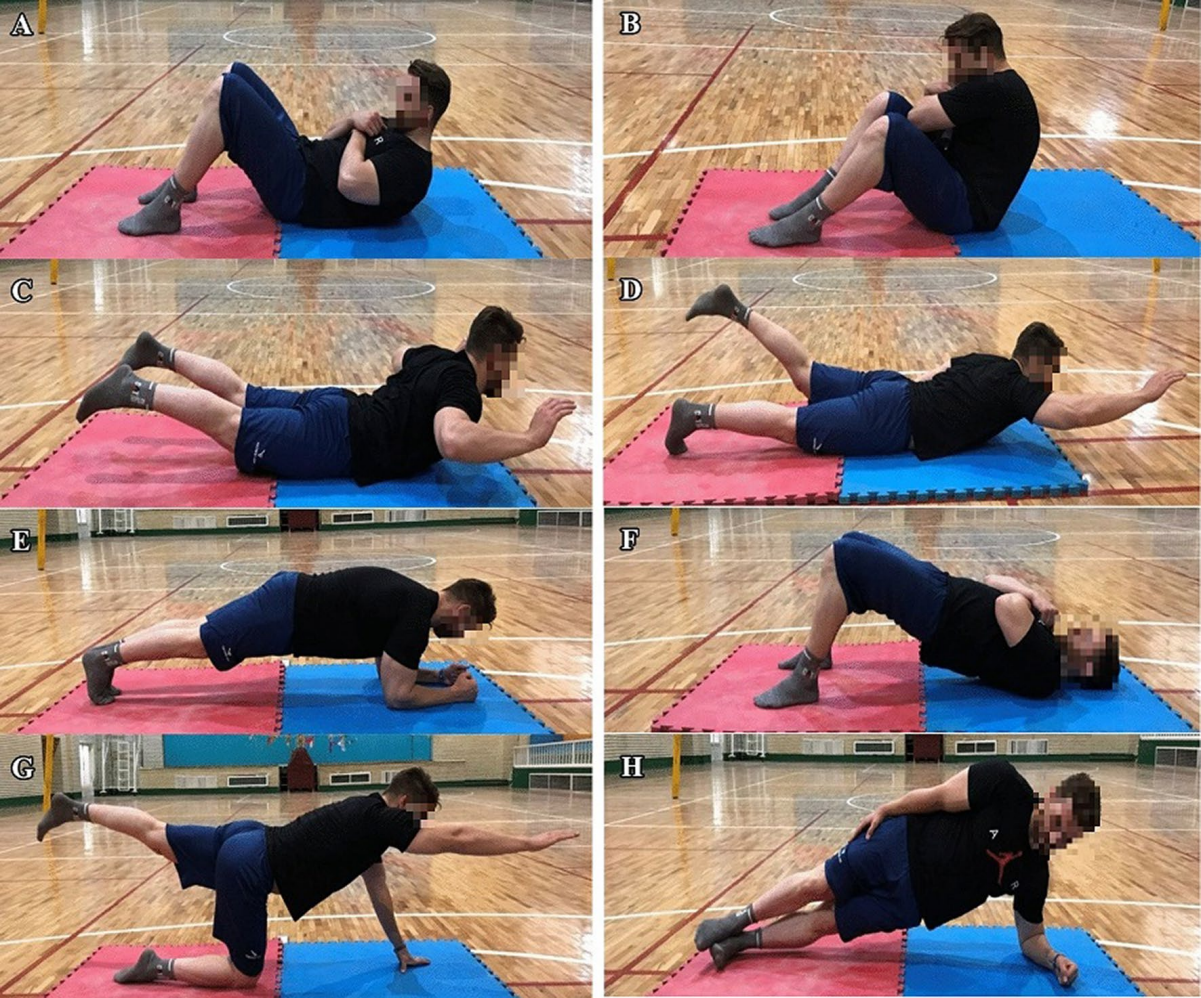
Core stability training program; Sit-up-1 (**A**), Sit-up-2 (**B**), Back extention-1 (**C**), Back extention-2 (**D**), Front plank (**E**), Back bridge (**F**), Quadruped (**G**), Side bridge (**H**).

The simple core stability training program consisted of 8 exercises (sit-up-1, sit-up-2, back extension-1, back extension-2, front plank, back bridge, quadruped exercise, and side bridge)^36^. The training group (n = 13) participated in the program three times a week for eight weeks, before starting their team training, under the supervision of a physical therapist. The training volume was 3 sets and the intensity increased from the 30 s to 60 s according to the degree of each participant’s achievement. Both groups continued their regular team schedule. At the beginning of each training session, general warm-up exercises were performed for both groups, for 10 min (Fig. 2).

### Statistical analysis

The sample size was determined using G-Power 3.1 software. Given an effect size of 0.37 based on the Biering-Sørensen test^37^, an alpha level of 0.05, and a power of 0.95, a minimum of 18 participants was needed for this study (9 individuals in each group). Expecting an attrision rate of 25%, 13 participants were considered for each group. All statistical analyses were performed in SPSS (ver. 24) with a confidence level of 95% and an alpha of ≤ 0.05. Shapiro–Wilk test was used for data distribution analysis. One-way analysis of covariance (ANCOVA), with a between-factor of the group (control, exercise) and participants' baseline scores included as a covariate, was used to determine if there were group differences in the outcomes at post-testing.

Based on the 95% confidence level in the current study, the minimal clinically important difference (MCID) was assessed using the distribution-based approach, while the minimal detectable change (MDC) was evaluated using the standard error of measurement (SEM) and the following equations:

$${\text{SEM }} = {\text{ SD}}_{{{\text{pre}}}} {\text{ }} \times \sqrt {\left( {1 - {\text{ rtest}}} \right)}$$

$${\text{MDC}}_{{\% 95}} = 1.96 \times \sqrt 2 \times {\text{ SEM}}$$

$${\text{MCID}}_{{{\text{RCI}}}} = 1.96 \times {\text{ SD}}_{{{\text{pre}}}} {\text{ }}\left[ {\sqrt {\left( {2 \times \left( {1 - {\text{rtest}}} \right)} \right)} } \right]$$

where rtest is intraclass correlation coefficient (ICC) and MCID_RCI_ is the minimal clinically important difference of reliable change index.

### Ethical approval

Ethics approval and consent to participate Written informed consent was obtained from the participants, and the patients give written informed consent for publication that was approved by the Ethics Committee of Hamedan University of Medical Sciences (code number: IR.UMSHA.REC.1396.840) and follows the guidelines of the declaration of Helsinki 2013.

## Results

The demographic characteristics of participants are presented in Table 1. There was no difference between the two groups with respect to the descriptive data (*P* ≥ 0.05).

### Knee kinematics

The ANCOVA results showed a significant difference between the two research groups in flexion and valgus angle in the post-test, after controlling the effect of the pre-test (covariate) (*P* < 0.05). The results showed that training group demonstrated higher flexion angle and lower valgus angle compared to the control group, at the post-testing time point. Knee valgus angle decreased by 63% for the training group and increased 22% for the control group over the eight weeks period (post-testing vs. baseline; Table 2).

**Table 2:** Effect of training on variables.

Variable	Groups	Pre-test (mean ± SD)	Post-test (mean ± SD)	Change Relative to Baselines § (%)	Between-groups	
					F	*P*
Knee kinematics						
Flexion angle	TG	59.9 ± 15.0	73.9 ± 13.0	23% ↑	5.92	0.02*
	CG	71.6 ± 13.8	72.1 ± 14.5	0.8% ↑		
Valgus angle	TG	6.9 ± 5.0	2.5 ± 3.2	63% ↓	24.05	< 0.01*
	CG	7.6 ± 7.8	9.3 ± 3.7	22% ↑		
Isometric hip muscle strength (base on %Kg)						
Hip extensors	TG	0.8 ± 0.1	0.8 ± 0.2	5% ↑	0.81	0.37
	CG	0.7 ± 0.2	0.7 ± 0.2	2% ↑		
Hip external rotators	TG	0.5 ± 0.1	0.7 ± 0.2	40% ↑	23.66	< 0.01*
	CG	0.5 ± 0.1	0.5 ± 0.2	0% ↑		
Hip abductors	TG	0.5 ± 0.1	0.6 ± 0.1	23% ↑	41.74	< 0.01*
	CG	0.5 ± 0.1	0.6 ± 0.0	4% ↑		
Core muscle endurance (base on seconds)						
Trunk flexion	TG	100.9 ± 20.6	143.4 ± 17.1	42% ↑	31.74	< 0.01*
	CG	98.8 ± 37.4	99.3 ± 29.6	0.5% ↑		
Biering-Sørensen	TG	96.1 ± 25.5	154.1 ± 22.4	60% ↑	86.94	< 0.01*
	CG	95.8 ± 28.5	89.3 ± 29.3	7% ↓		
Right-side plank	TG	49.1 ± 7.4	79.6 ± 11.9	62% ↑	79.60	< 0.01*
	CG	45.4 ± 8.3	43.7 ± 10.1	2% ↑		
Left-side plank	TG	45.4 ± 6.7	71.8 ± 9.9	58% ↑	62.52	< 0.01*
	CG	42.3 ± 5.3	41.1 ± 8.9	3% ↓		

TG = Training Group; CG = Control Group.

^§^, Percent change (↓decrease, ↑ increase).

*, Significant.

### Isometric hip muscle strength

The ANCOVA results showed a significant difference between the two groups in hip external rotators and abductors in the post-test (*P* < 0.05). No significant differences were observed between hip extensors strengths in the post-test (*P* > 0.05). Training group demonstrated higher hip external rotators and abductors strength compared to the control group at the post-testing. The strength of the hip external rotators, and abductors for the training group increased by 40%, and 23% respectively, and for control group increased by 0%, and 4%; over the eight weeks period (post-testing vs. baseline). As a result, the training group demonstrated higher hip strength compared to the control group (Table 2).

### Core muscle endurance

The ANCOVA results showed a significant difference between the two groups for trunk flexion endurance, Biering-Sørensen test, right-side plank, and left-side plank in the post-test (*P* < 0.05). Training group demonstrated higher core muscle endurance compared to the control group. Post-testing trunk flexion endurance (42%), Biering-Sørensen (60%), right-side plank (62%), and left-side plank (58%) increased for the training group over the eight weeks period (post-testing vs. baseline; Table 2).

It was observed that the mean pre/post difference in variables of the knee kinematics, isometric hip muscle strength (except hip extensors), and core muscle endurance (except trunk flexion) test exceeded both MDC and MCID values. This indicates that in addition to the changes being statistically significant, the value of changes obtained is also clinically significant (Table 3).

**Table 3:** The MDC and MCID values of the study variables.

Variable	SD_pre_ TG	Δ Score	SEM	MDC_%95_	MCID_RCI_
Flexion angle	15.02	14.03	2.10	5.80	5.90
Valgus angle	5.02	4.34	1.20	3.31	3.44
Hip extensors (% Kg)	0.14	0.04	0.1	0.27	0.08
Hip external rotators (% Kg)	0.11	0.19	0.03	0.08	0.09
Hip abductors (% Kg)	0.07	0.12	0.026	0.05	0.07
Trunk flexion Endurance (s)	20.58	42.46	20	55.27	33.07
Biering-Sørensen (s)	25.54	57.92	6.64	18.35	18.52
Right-side plank (s)	7.43	30.54	1.63	4.5	4.7
Left-side plank (s)	6.75	26.46	1.48	4.09	4.23

## Discussion

The purpose of our study was to investigate the effects of eight weeks of simple core stability training on improving knee kinematics, hip strength, and trunk endurance in male athletes who had undergone ACL reconstruction and completed conventional post-operative rehabilitation. The finding of this study showed that eight weeks of core stability training resulted in a significant change in core muscle endurance, knee kinematics, and isometric hip muscle strength (except for hip extensor strength) between the pre-test and the post-test (*p* < 0.05) in male athletes with a history of ACLR.

Improving the efficiency of core muscles to stabilize trunk at different planes can be effective in reducing the risk of non-contact ACL injury in athletes^38^. Based on a study, the endurance of core muscles is more important than their maximum strength because 55% to 58% of the abdominal muscle fibers are made up of type I fibers^39^. Facilitating the simultaneous contraction of muscles around lumbar vertebrae (such as abdominal and oblique, transverse abdominis, multifidus, and erector spine muscles) may increase the stability of the spine, pelvis during functional movements which can be valuable for athletes^40^. The core muscles are the center of our body and it functions to stabilize the trunk while the upper and lower limbs move during functional movements^41,42^. Therefore, it seems that weak core muscles cause an interruption in energy transfer and create abnormal movement patterns, which increases the probability of injury^11,43,44^. A more stable core allows for more efficient distal segment movements and protects the distal joints^45,46^. When this system works properly, it leads to proper distribution and maximum force generation with minimal compressive, translational, and shearing forces in the joints of the kinetic chain, as well as optimal control of movements and proper absorption of shock forces caused by ground reaction forces during landing^47^. A strong trunk provides a stable base and structure to generate torques created in the limbs^48^. According to a study, the more the core was strengthened, the smaller knee valgus angle at initial contact during the cutting task^49^. Wilson et al.(2006) reported that participants with greater isometric core strength demonstrated lower knee valgus angle during single-leg squat^50^. Our findings agree with the observations of these studies. Our results show that core stability training with increasing core endurance, decreasing knee valgus, and increasing knee flexion helps to reduce the risk of ACL re-injury in athletes with ACLR. Considering that an increased valgus angle and decreased knee flexion are associated with an increased risk of ACL injuries, increasing the endurance of core muscles can decrease the risk of ACL injury by controlling the lower limb kinematics^51–53^. Also, strengthening the core stability muscles directly or indirectly affects the strength of lower extremity muscles because in the kinetic chain, local control of the vertebrae, lumbopelvic control, and positional control interact with each other^54^. Therefore, it seems that core stability training used in this study can increase the stability of trunk and pelvis by increasing the endurance of the core muscles, this causes the pelvis to have no extra movements and stabilizes the movements in the lower joints (*e.g.* the knees).

Due to the high rate of ACL re-injury, tertiary prevention programs should be designed which are applicable in different places and can be done in a limited amount of time so that they do not interfere with the main training of sports teams. According to the results of this study, it is suggested that the proposed training protocol be used in the daily warm-up program of athletes because these exercises (which are a selection of effective core stability exercises) can be performed in a short and limited time. However, further RCTs with sufficient follow-up should investigate if enhanced core muscle strength and endurance can really contribute to reduced re-/injury risk reduction.

One of the limitations of this study was the lack of comparison of injured and healthy limbs of athletes, which is suggested to be considered in future studies. Also, the absence of a control group consisting of non-injured athletes was another limitation of this study. Thus, further studies are needed to compare ACLR athletes with non-injured athletes. The present study was performed on ACLR athletes with hamstring tendon autograft and the results are generalizable to this population. In addition, these results may vary in athletes with concurrent ligament injuries. Hence, it is recommended that multiple injury studies be conducted. Finally, further studies with sufficient follow-up should investigate whether increasing core muscle strength and endurance can really contribute to reduced re-/injury risk reduction.

## Conclusion

The results of this study showed that eight weeks of simple core stability training improves core muscle endurance, hip abductor and external rotator strength, and knee kinematics in male athletes who had undergone ACL reconstruction and completed conventional post-operative rehabilitation. Based on the findings and results of the present study, performing simple core stability training prior to team routine training could reduce the risk of secondary injury. It is recommended that trainers and physical therapists use core stability training in designing exercises, prevention protocols, and regular rehabilitation schemes.

## Data Availability

The datasets collected during the current study are available from the corresponding author upon reasonable request.

## Abbreviations

ACLR: Anterior cruciate ligament reconstruction
ACL: Anterior cruciate ligament
TG: Training group
CG: Control group
ANCOVA: Analysis of covariance
KAA: Knee abduction angle
DKV: Dynamic knee valgus
IRB: Institutional review board
BMI: Body mass index
ASIS: Anterior superior iliac spine
